# Editorial: Diversity and Universality in Causal Cognition

**DOI:** 10.3389/fpsyg.2017.01767

**Published:** 2017-10-10

**Authors:** Sieghard Beller, Andrea Bender, Michael R. Waldmann

**Affiliations:** ^1^Department of Psychosocial Science, University of Bergen, Bergen, Norway; ^2^Department of Psychology, University of Göttingen, Göttingen, Germany

**Keywords:** causal cognition, agency, culture, language, methods, interdisciplinary approach

The capacity to acquire and use causal knowledge belongs to the central cognitive competencies that allow us to orient in the world, and this knowledge shapes our cognitive, affective, and behavioral responses. Its central role renders causal cognition a core topic for the social and cognitive sciences. But is causal cognition a universal and uniform phenomenon, or are there cultural differences in the way people represent the causal texture of the world? In spite of extensive research on causal cognition in the past decades (Waldmann, 2017), little is known about cultural diversity in how people perceive, represent, and reason about causal relationships (Bender et al., 2017). The main goal of this research topic is therefore to compile evidence for both diversity and universality in causal cognition, with the aim of pushing the field forward.

One set of the contributions to this topic addresses questions revolving around *people's conceptualization of causality and agency*, with a focus on situations that involve a human agent. To this end, Le Guen et al. investigate how rural Mayan Yucatec and Tseltal speakers from Mexico and urban students from Mexico and Germany account for events for which the relations between intention, action, and outcome are varied. The groups converge in recognizing explicit links between actions and outcomes as causal, but differ in how they interpret non-law-like relations. Specifically, the notion of “chance” proved sensitive to task characteristics, cultural background, and language used.

Another topic that has attracted interest is the phenomenon of “causal deviance,” which refers to situations in which an outcome satisfies an agent's intention, but is not brought about by this agent's action. For such cases, studies with US American participants have repeatedly reported a higher readiness to attribute intentionality to *immoral* than to *amoral* actions. For example, in a causally deviant situation the amoral action of “hitting a bull's eye” is not considered intentional in contrast to the immoral action of “hitting the aunt's heart.” Seeking a more fine-grained understanding of this phenomenon, Sousa et al. find the asymmetry to be fairly robust across varying degrees of causal deviance, even if mediated by judgments of action and blame, which they interpret as evidence for the existence of multiple concepts linked to intentional action.

While these authors consider intentionality to be a basic and universal concept, Astuti and Bloch explore the possibility of cross-cultural variation by investigating the extent to which Malagasy people take intentionality into account when assessing acts of wrongdoing. They conclude that, while intentionality is indeed considered important in mundane cases of wrongdoing, its relevance decreases for events with more severe consequences for society, thereby pointing at both cross-cultural commonalities and differences (for a continuation of the debate, see also Sousa and Swiney, 2016).

A further factor that may tune people's attention to agency and intentionality is *language*. Agency information can be encoded in different ways, for instance through word order, case marking, or verb type. That these linguistic cues affect the assignment of causal roles has been demonstrated by Bender and Beller with speakers of German and Tongan.

An important area of causal cognition is *reasoning about complex systems*. Research reviewed here focuses on cases of economic decision-making, complex problem solving, and ethnomedical beliefs. In the first of these studies, Tucker et al. investigate the causal models Malagasy farmers, foragers, and fishermen use when explaining success and failure. Tucker and colleagues find that biological and economic events are attributed primarily to natural causes, whereas individuals' success and failure tend to be attributed to “supernatural” factors. As natural and supernatural factors represent distinct sets within a single explanatory framework—with the supernatural forces driving the natural ones—the Malagasy data suggest a type of “integrative thinking” that the authors consider to be common in unpredictable environments. A suitable context for testing this hypothesis is the large-scale project described in Bennardo's contribution, which seeks to identify the main causal forces in cultural models of nature across a broad range of populations.

Both economic decisions under uncertainty and cognitive models of nature are paradigmatic test cases for investigating causal reasoning about *complex systems*. Simulations of systems (*microworlds*) are used to study complex problem solving, with participants being responsible for retaining a balance between several interconnected factors. Complex systems are characterized by non-transparent relations and non-linear processes, which pose substantial challenges for problem-solving and management (Funke). Because successful problem-solving typically involves updating a cognitive model of the interactions, microworlds can be used to diagnose causal perception, reasoning, understanding, and intervention. As argued by Güss and Robinson, participants' models and strategies may be affected by cultural background on several levels: knowledge, problem-solving heuristics, and perceptions of control by culturally mediated experiences; priorities in problem-solving by culture-specific values; and the temporal horizon for planning and decision-making by the cultural learning environment. To what extent microworlds are useful for cross-cultural research, whether problems of different complexity require different types of causal cognition, or whether they constitute qualitatively different phenomena is discussed both within the research topic (Funke; Greiff and Martin) and beyond (Dörner and Funke, 2017).

A particularly relevant example of reasoning about complex causal relations is the diagnosis of mental disorders. Taking causal model theory as the starting point, Hagmayer and Engelmann derive predictions for systems of causal beliefs, applied here to lay theories of depression. Their analysis of data from a systematic literature review reveals cross-cultural convergence about relevant observable causes (e.g., stress), but substantially less cross-cultural agreement for hidden, especially supernatural causes.

The third set of contributions to the present research topic addresses *methodological problems* typically encountered in cross-cultural research, and discusses possible solutions and their relevance for theoretical advances in the field. Beer and Bender investigate how people in an unfamiliar socio-cultural setting account for the behavior of others conditional upon their category membership. Setting off as an attempt to explore information search strategies among the Wampar in Papua New Guinea, the contribution turns into a discussion of the difficulties with parallelizing cognitive tasks across cultures.

Not only designing new tasks for cross-cultural investigations of causal cognition is challenging—even the attempt to interpret available evidence is tricky. Ethnographic fieldwork has gathered a plethora of potentially relevant data that can be reconstructed as examples of causal reasoning (e.g., reasoning about witchcraft). However, in these studies the data are often not described in terms of abstract causal theories. Thus, relevant information is hard to localize and difficult to identify as relevant. How ethnographic descriptions can still be used to investigate causal reasoning is laid out by Widlok, pointing to culture-specific notions of time and extensions of personhood and agency as essential components of causal understanding (see also Peeters, 2015).

The malleability of cultural perspectives over time and the inalienability of contextual information, a critical point raised by Widlok, is emphasized further by Iliev and ojalehto who call for diachronic analyses within single cultures as an essential complement to synchronic investigations across cultures. They introduce automated text analyses as a valuable tool for tracking how the concern with causality, the usage of causal vocabulary, and causal concepts themselves have changed over time.

Extending this historic perspective to the evolutionary roots of causal cognition, Haidle scrutinizes archaeological findings as evidence of causal cognition in our ancestors. Based on the idea that tool construction presupposes considerations of cause-effect relations, she uses data on the composition of tool remains to infer, by way of reverse-engineering, which components of causal cognition allowed our ancestors to invent these tools.

Finally, Kronenfeld in his theoretical piece reverts the usual reading of *causal cognition* to explore possible ways in which cognition may be considered causal, focusing in particular on collective practices.

In summary, the 15 contributions to this research topic address a broad range of aspects of causal cognition: from perceptions and representations of causal relations through judgments of blameworthiness and punishment to ways in which illnesses are explained and treated. The articles describe approaches from a broad range of disciplines—including anthropology, archaeology, linguistics, philosophy, and psychology—and provide evidence for both the universality and diversity of causal cognition. Jointly, they support the assumption that core components of causal cognition are widely shared across historic and cultural contexts, but are also refined, shaped, and occasionally altered through processes of cultural elaboration and transmission that are characteristic for our species. Thus, these contributions highlight the need for more in-depth investigations of the cultural impacts in this domain, preferably through concerted efforts across disciplines, timescales, and levels of analyses (Bender and Beller, 2016).

## Author contributions

All authors listed have made a substantial and direct intellectual contribution to the work and approved it for publication.

### Conflict of interest statement

The authors declare that the research was conducted in the absence of any commercial or financial relationships that could be construed as a potential conflict of interest.
